# Response of *Posidonia oceanica* seagrass and its epibiont communities to ocean acidification

**DOI:** 10.1371/journal.pone.0181531

**Published:** 2017-08-09

**Authors:** Katja Guilini, Miriam Weber, Dirk de Beer, Matthias Schneider, Massimiliano Molari, Christian Lott, Wanda Bodnar, Thibaud Mascart, Marleen De Troch, Ann Vanreusel

**Affiliations:** 1 Marine Biology Research Group, Department of Biology, Ghent University, Ghent, Belgium; 2 HGF-MPG Joint Research Group on Deep Sea Ecology and Technology, Max Planck Institute for Marine Microbiology, Bremen, Germany; 3 HYDRA Institute for Marine Sciences, Elba Field Station, Italy; 4 Microsensor Group, Max Planck Institute for Marine Microbiology, Bremen, Germany; University of Alabama, UNITED STATES

## Abstract

The unprecedented rate of CO_2_ increase in our atmosphere and subsequent ocean acidification (OA) threatens coastal ecosystems. To forecast the functioning of coastal seagrass ecosystems in acidified oceans, more knowledge on the long-term adaptive capacities of seagrass species and their epibionts is needed. Therefore we studied morphological characteristics of *Posidonia oceanica* and the structure of its epibiont communities at a Mediterranean volcanic CO_2_ vent off Panarea Island (Italy) and performed a laboratory experiment to test the effect of OA on *P*. *oceanica* photosynthesis and its potential buffering capacity. At the study site east of Basiluzzo Islet, venting of CO_2_ gas was controlled by tides, resulting in an average pH difference of 0.1 between the vent and reference site. *P*. *oceanica* shoot and leaf density was unaffected by these levels of OA, although shorter leaves at the vent site suggest increased susceptibility to erosion, potentially by herbivores. The community of sessile epibionts differed in composition and was characterized by a higher species richness at the vent site, though net epiphytic calcium carbonate concentration was similar. These findings suggest a higher ecosystem complexity at the vent site, which may have facilitated the higher diversity of copepods in the otherwise unaffected motile epibiont community. In the laboratory experiment, *P*. *oceanica* photosynthesis increased with decreasing pH_T_ (7.6, 6.6, 5.5), which induced an elevated pH at the leaf surfaces of up to 0.5 units compared to the ambient seawater pH_T_ of 6.6. This suggests a temporary pH buffering in the diffusive boundary layer of leaves, which could be favorable for epibiont organisms. The results of this multispecies study contribute to understanding community-level responses and underlying processes in long-term acidified conditions. Increased replication and monitoring of physico-chemical parameters on an annual scale are, however, recommended to assure that the biological responses observed during a short period reflect long-term dynamics of these parameters.

## Introduction

Seagrasses, which are considered to be amongst the most productive ecosystems worldwide also support high biodiversity [1]. Hence, their current worldwide decline at 7% loss per year is worrying [2,3]. Major threats to seagrass ecosystems and the services they provide include the immediate impacts of coastal development by growing human populations and the indirect impacts of the unprecedented increase in atmospheric CO_2_ concentrations through global warming [3] and ocean acidification [4]. Evaluating the response of seagrass ecosystems to pressing environmental stressors is crucial for effective management of coastal regions in the future.

Human induced increases in atmospheric CO_2_ concentrations result in changes in ocean carbonate chemistry and pH, a process termed ocean acidification (OA). OA raises concerns about the health of ocean ecosystems, particularly the viability of calcifying organisms to cope with predicted seawater pH decreases of 0.06–0.32 units by the year 2100, depending on the considered CO_2_ emission scenario [5]. The sole increase in partial pressure of CO_2_ (*p*CO_2_), however, is not expected to have a direct negative impact on seagrasses. On the contrary, it has been observed that when exposed to increased *p*CO_2_, seagrasses increase their photosynthetic rates [6–13], shoot productivity [14], density [15], leaf growth rates [8,16], flowering frequency [17], and biomass [6,11,15,18]. Environmental stressors such as nitrogen limitation [9] or an increase in turbidity [19,20], temperature [21–23] and grazing [24] that occur simultaneously, have been shown to counteract the direct positive effects on seagrasses. Moreover, non-calcifying epiphytes may respond positively to increased *p*CO_2_ and cause shading [25,26], while calcifying epiphyte coverage might decline under similar conditions and induce high light exposure [4]. The reaction of epibiota generally depends on its ability to cope with a change in the inherent natural diurnal variability in seawater pH created by seagrass metabolism (ΔpH range: 0.06 to >1; [27–31]). Overall, it seems that the global effect of increases in *p*CO_2_ on seagrasses may be spatially heterogeneous, species-specific and related to the plant physiology structure [31]. Particularly due to effects being dependent on geochemical site characteristics and resulting from the interplay of several physico-chemical stressors and interactions with other biota [9,12].

To forecast the functioning of marine seagrass ecosystems in acidified oceans, we need to increase our knowledge on the long-term adaptive capacities of different seagrass species to high *p*CO_2_ conditions, while taking into account the physico-chemical settings and their associated biota. Volcanic submarine CO_2_ vents may act as natural analogues to study the effects of long-term exposure to acidified seawater in a holistic approach. However, the high spatial and temporal variability in CO_2_ and the interference of other variables that may differ across the same gradient could hamper the detection of a reliable dose-response relationship [14,18,31,32]. More observations at different CO_2_ vent systems may, however, facilitate our understanding, particularly when these studies also consider laboratory and in situ pH manipulations that examine the same ecosystem-level measures.

Only a limited number of seagrass species have been examined at natural CO_2_ vents (i.e. *Cymodocea nodosa*, *C*. *serrulata*, *Halophila ovalis*, *Posidionia oceanica*) and these studies primarily investigated the effects on seagrass productivity and growth response [12,19,24]. Some studies have taken into account the effects on the associated sessile epiphytes, bryozoans and hydrozoans, the macro-invertebrates and the motile megafaunal grazers [4,14,18,33–36]. The response of motile meiofauna living in high abundances on the leaves or the vertical rhizomes [37] is still unexplored. Despite the valuable information previous CO_2_ vent studies have provided, sampling different biological elements at sites characterized by variable biogeochemistry (e.g. pH ranges) and sampling at different times, hampers the integration of data. This in turn hinders the interpretation of potential interacting processes and cascading responses over different levels of organization. It is therefore advisable to perform multispecies studies which aid in unraveling community-level responses and their underlying processes, permitting our comprehension of the populations’ ability to adapt and evolve [38].

**Fig 1:**
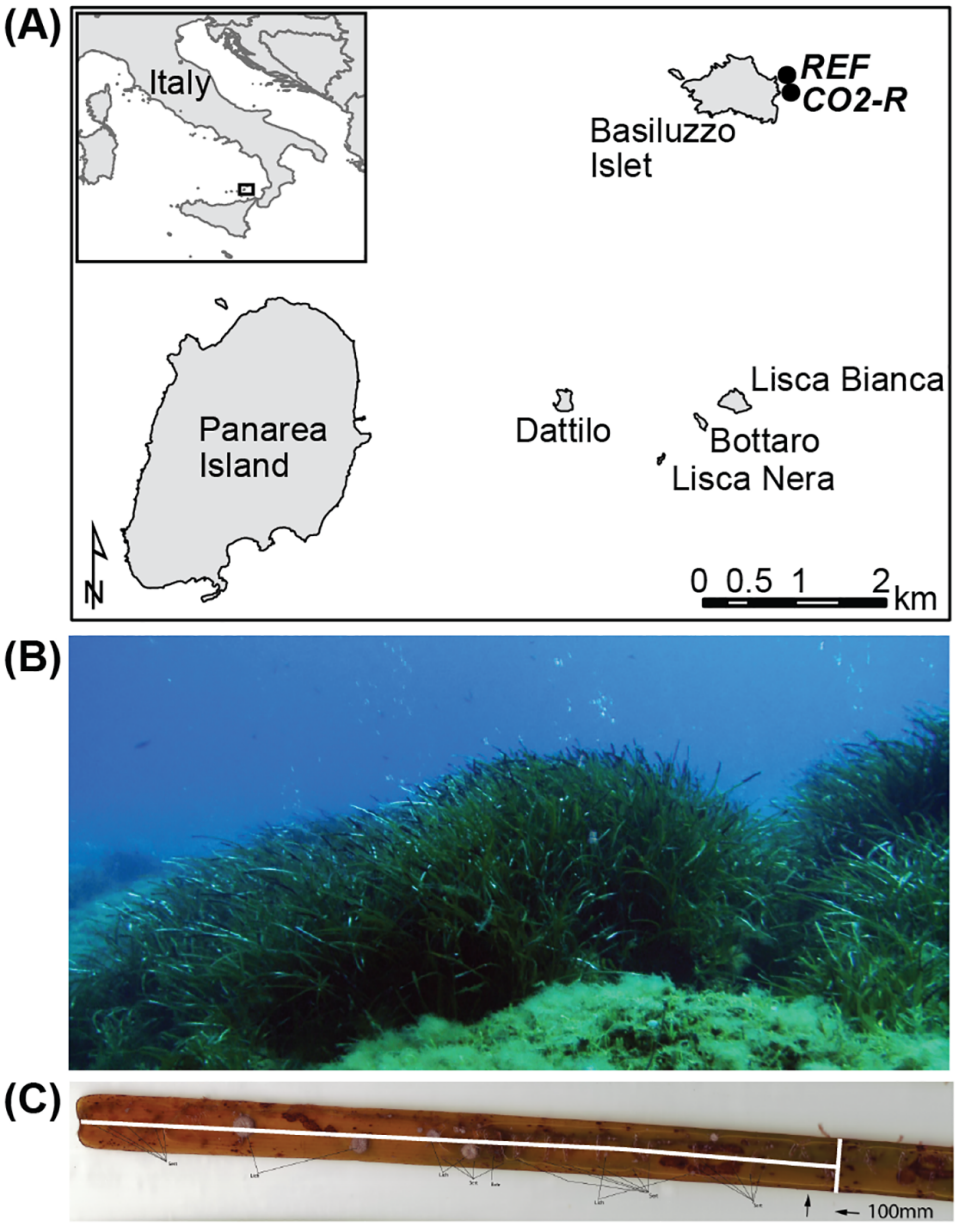
Location and illustration of the study area near Basiluzzo Islet (Panarea, Italy) and an example of a *P*. *oceanica* leaf tip. (A) Map indicating the location of the two study sites east of Basiluzzo Islet (Mediterranean Sea): the reference site ‘REF’ (38°39.827’ N, 15°07.118’ E) and the CO_2_ vent site ‘CO2-R’ (38°39.749’ N, 15°07.132’ E). Software: ESRI—ArcGIS Desktop 10.1, map layers originating from ESRI—Europe—world countries 2008 and ESRI—ArcGIS online (layer package by fraciccio67)–comuni, (http://www.esri.com). (B) Overview of a *P*. *oceanica* seagrass meadow at CO2-R with visible gas bubbling. (C) *P*. *oceanica* leaf tip with indication of the examined part, i.e. half of the upper 10 cm of the leaf.

In this study, we investigated the Mediterranean seagrass *Posidonia oceanica* (L.) Delile and its associated epibionts at a CO_2_ vent area in the vicinity of Bassiluzo Islet, off Panarea Island (Italy, Fig 1). Studies at complex natural CO_2_ vent environments require a description of the abiotic settings and dynamics. We therefore analyzed seawater and pore water from different heights above and below the surface of the seagrass beds and logged the diurnal variation in seawater pH, O_2_, water depth and oxygen reduction potential. *P*. *oceanica* and its associated sessile and motile epibionts were investigated by testing the following hypotheses: (H_1_) Long-term acidified seawater conditions result in an increase in *P*. *oceanica* leaf and shoot densities and leaf length. On the contrary, (H_2_) epibiont communities on *P*. *oceanica* are vulnerable to long-term acidified seawater conditions, and exhibit altered community structures and a reduction in abundances and diversities. Additionally, we performed a laboratory experiment to measure the potential buffering capacity of seagrass leaves and test if an increase in *p*CO_2_ enhances the photosynthetic rate of *P*. *oceanica* (H_3_).

## Materials and methods

### Study site

The study area was located east of Basiluzzo Islet close to Panarea Island in the Mediterranean Sea (Fig 1). The area consists of patchily distributed seagrass meadows, typical sandy sediments and rocky outcrops. It covers locations where fluids and gases rich in CO_2_ (ca. 97%) [39] escape the seabed as a result of volcanic activity [40], as well as reference sites without any measurable evidence of seepage. To determine the biogeochemical characteristics of the seagrass meadow environment, sensors were used in situ and samples were obtained by SCUBA diving during the sampling campaign in 2012 (02.06–12.06). An exception is the pore water samples which were obtained in 2013 (01.06–14.06). Two sites (200 m apart) were the focus of this study: one with red sediment where evenly distributed gas seepage occurred (“CO2-R”, 38°39.749’ N, 15°07.132’ E, 13–14 m water depth) and a second site where sediments were colored grey and no gas seepage occurred (“REF”, 38°39.827’ N, 15°07.118’ E, 14–15 m water depth). Both study sites covered approximately 50 x 50 m. Water temperature at 1 m above the seafloor (asf) was 18.7 to 19.3°C, and *P*. *oceanica* meadows occurred in numerous patches at both sites. Sampling was performed after receiving permits of the Soprintendenza del Mare, Assessorato dei Beni culturali e del’Identità siciliana, Regione Sicilia, Palermo, Italy.

### Observational study

#### Seawater and pore water

RBR-Dataloggers (XR-420 D; Ottawa, Canada) measured and logged pH_NBS_, O_2_, ORP (oxygen reduction potential) and pressure (tides) every three seconds, continuously for 15 days, in the midst of a seagrass meadow (at 2 cm asf) at both sites. These data series were averaged per 5 minutes. Additionally, seawater was sampled at 80 cm and 10 cm asf, i.e. above and within each seagrass patch, with a 5 L-Niskin bottle and glass syringes, respectively. Pore water was sampled with a stainless steel lancet and attached syringe with filter (22 μm) at 0 cm, -10 cm and -20 cm asf in the meadows. Triplicate seawater and pore water samples were collected on one occasion only and might therefore mask diurnal variation. Upon return to the laboratory, sub-samples were taken from the collected seawater samples using 10 mL syringes, attached to Rhizons MOM (19.21.21F, pore size 0.15 μm; Rhizosphere Research Products, Wageningen, Netherlands) to determine dissolved inorganic carbon (DIC), pH_NBS_, total alkalinity (TA), and nutrient concentrations (NH_4_^+^, PO_4_^3-^, NO_2_^-^, NO_3_^-^ + NO_2_^-^, Si). Dissolved silicate was measured photometrically according to Grasshoff et al. [41]. NH_4_^+^, PO_4_^3-^, NO_2_^-^, NO_3_^-^ were measured spectrophotometrically with a continuous-flow analyzer (Bran & Lübbe GmbH, Norderstedt, Germany) using a variant of the method of Grasshoff et al. [41]. Measurements of pH_NBS_ were directly done in the field with a pH 96 by WTW (Wissenschaftlich-Technische Werkstätten GmbH, Weilheim, Germany) and an InLab Semi-Micro electrode by Mettler Toledo (Gießen, Germany). The probes were calibrated at ambient temperature with conventional buffer solutions by Mettler Toledo (pH 4.00 and 7.00). All pH_NBS_ values were converted to pH values on the total scale (pH_T_), via a conversion from pH_NBS_ to pH on seawater scale (pH_SWS_) according to Lewis & Wallace [42]: *a*_H_ = 10^(-pHNBS)^ = *f*_H_ * H_SWS_, where *a*_H_ is the activity and *f*_H_ is the activity coefficient of the H^+^ ion. Therefore, pH_NBS_ = pH_SWS_—log_10_ *f*_H_. Takahashi et al. [43] provides a formula for the activity coefficient *f*_H_ as a function of salinity (S) and temperature (T): *f*_H_ = 1.2948–0.002036 * T + (0.0004607–0.000001475 * T) * S^2^. Finally, the conversion from pH_SWS_ to pH_T_ is described in Zeebe & Wolf-Gladrow [44]. For DIC and TA, 2 mL seawater was stored into headspace-free glass vials at 4°C. DIC and TA were assessed via flow injection analysis [45] and two-point titration [46], respectively. Degree of saturation for calcite and aragonite (Ωcal and Ωar) were calculated based on TA and DIC data and in situ temperature and salinity conditions using the R package seacarb v 3.0.11 [47]. The carbonate dissociation constants (K_1_ and K_2_) used were those of Roy et al. [48], K_f_ from Dickson and Riley [49] recommended by Dickson and Goyet [50] and K_s_ from Dickson [51]. Average pH values were calculated based on averaged H^+^ concentrations and accompanied by their coefficient of variation (c.v.).

### *P*. *oceanica* growth characteristics

SCUBA divers determined shoot density at replicated patches (n = 3) at both sites, using a circular metal frame with a diameter of 56.4 cm (i.e. 0.25 m^2^). After each count, the leaves of 10 shoots were randomly sampled (i.e. along a line each 5 cm, insuring low probability to match any natural pattern) by cutting them off at the leaf bases. All leaves of one shoot were carefully transferred into a plastic bag, sealed and transported. In the laboratory, the number of leaves per shoot were counted, both sides of all leaves were scanned wet in high resolution using a flatbed scanner to facilitate length and area measurements of each leaf using the image editing software ImageJ [52] and to also document epibiotic species. Subsequently, the leaves were stored in a 4% formaldehyde-seawater solution. The leaf area indices are found by multiplying the average leaf area per shoot by the estimated shoot density of each individual sample [53].

### Epiphytic calcium carbonate (CaCO_3_) weight

Seagrass leaves (n = 5) from both sites were sampled and scanned as described before. The upper 5 cm were cut off, air dried and weighed before being washed in 0.1 M HCl. After all CaCO_3_ was dissolved, the leaves were air dried again and weighed to determine the CaCO_3_ weight per leaf area (mg cm^-2^).

### Sessile epibiont community

From the 10 shoots that were collected per quadrat (n = 3), 5 were randomly chosen and used to examine the epibiotic community (i.e. 15 shoots site^-1^). Epibiota on the upper 10 cm of each leaf tip and on half of the area of the concave leaf side were analyzed (Fig 1). Only intact leaf tips were considered for epibiont abundance. All sessile organisms were identified to the most practical taxonomic level based on the high resolution scans of the fresh material and the preserved sample, using a microscope (5x10x). Biota or colonies with more than 1 mm in diameter or length were considered. Additionally, bite marks of macro-herbivores were recorded and quantified on all leaves from the 5 shoots, to estimate seagrass consumption. Three main consumers can be distinguished by their bite marks: the isopod *Idotea* sp. (Crustacea, Isopoda), the sea urchin *Paracentrotus lividus* (Echinodermata, Echinoidea), and the fish *Sarpa salpa* (Actinopterygii, Sparidae). The total number of leaves examined per site was influenced by the number of leaves per shoots (CO2-R: 195, REF: 165). Bite mark abundance was considered as number of bite marks per leaf.

### Motile epibiont (meiofauna) community

Three replicate seagrass patches per site were sampled to study the meiofauna associated with seagrass leaves and vertical rhizomes, i.e. two subunits of the plants distinguished based on microhabitat structure [37]. Approximately 12 to 18 leaves per sample were collected by placing a plastic bag over the leaves and gently cutting the leaves at the base before closing the bags with elastic bands. The remaining vertical rhizomes, which included the degrading leaf sheaths, were cut off and gently transferred into separate plastic bags, to minimize transfer through the water column. Upon return to the laboratory, both leaf and vertical rhizomes (further referred to as rhizomes) samples were sieved (32 μm) to eliminate the water. The retained material was stored in a 4% formaldehyde-seawater solution. Samples were then emptied over stacked 1 mm and 32 μm sieves and rinsed thoroughly. Meiofauna (size class 0.032–1 mm) were extracted from the samples through triple density centrifugation with the colloidal silica polymer LUDOX TM 40 (ρ: 1.18). All metazoan meiofauna were classified at higher taxon levels and counted under a Leica stereoscopic microscope (16x5x). ImageJ software was used to estimate the surface of scanned leaves to standardize meiofauna densities on the leaves (individuals 100 cm^-2^). To standardize the meiofauna densities on the rhizomes (individuals 10 cm^-3^), the volume of rhizomes was determined by the amount of water displaced when the rhizomes were submerged. Whenever possible, 120 copepods and 100 nematodes were randomly handpicked with a needle before identification to species (numeric) level. Diversity of the meiofauna at a higher taxon level and at nematode and copepod species level were assessed in terms of the Hill’s indices (H_0_, H_1_, H_2_, H_inf_; [54]). The indices are sample-size sensitive and therefore analysed for leaves and rhizomes separately. The rarefaction index (ET(51) or ES(51); [55]), which is independent of sample size, was also calculated and analysed together for both rhizomes and leaves. Based on nematode mouth morphology, all identified individuals were classified in functional feeding groups according to Wieser [56]. Nematode functional diversity was assessed by calculating the inverse trophic index (1/TI or 1/Σθ^2^, where θ is the percentage of each feeding group; [54]).

### Laboratory experiments

Seagrass shoots were collected by SCUBA divers at both sites and stored on shore in aerated tanks in seawater at in-situ conditions. Measurements were performed within 2 days after collection. For laboratory experiments, one shoot, that consisted of intact leaves and vertical rhizomes, was placed in a flow cell (volume: 1 L). Seawater, from the site of the plant’s origin, was pumped from an aerated recirculation tank (volume: 20 L) through the flow cell. One leaf was placed on two stoppers that were 5 to 8 cm apart and held in place with two small stoppers. Thus both sides of the leaf were freely exposed to the flowing water. Seawater temperature was held constant at 22°C and flow velocity was maintained at ca. 5 cm s^-1^. The light source was a fiber optic halogen lamp (Schott KL1500, Germany). Incident light intensity was 200 μmol photons m^-2^ s^-1^, quantified as scalar irradiance with a Biospherical Instruments meter (QSL-100, USA). Liquid membrane type pH microsensors and potentiometric O_2_ microsensors were prepared, calibrated and applied as described by Revsbech [57] and de Beer [58]. The microsensors had a tip size of 10 μm and response times (t90) of less than 1 s. Profiles were measured by positioning the microsensors using a motorized micromanipulator. Net photosynthesis was determined as the oxygen flux (J) from the leaf surface, calculated from the interfacial oxygen gradient (dC/dX) using Fick's 1. law: J = D*dC/dX, where D is the diffusion coefficient for oxygen (i.e. 2.16 x 10^−9^ m^2^ s^-1^ at 22°C, salinity of 40). Net photosynthesis rates were determined on three leaves that were each exposed to pH_T_ 7.6, 6.6 and 5.5, for a few hours per pH regime. The pH was reduced and stabilized by flushing the recirculation tank with CO_2_ gas and at each pH, 5 to 10 profiles were measured on the convex side of a leaf and averaged, after the shoots acclimatized to the manipulated conditions for 1 to 2 hours. Likewise, pH profiles were measured over a distance of 0 to 1000 μm off the leaves’ surface in both light (n = 2–3 for REF, n = 3 for CO2-R) and dark (n = 1 for REF) conditions at each pH. The pH in the flow cell was constantly monitored with a pH sensor and kept stable within 0.1 unit. All pH_NBS_ values were converted to pH_T_ as mentioned earlier and average pH_T_ values were calculated based on averaged H^+^ concentrations and accompanied by their variation (c.v.).

### Statistical analysis

To characterize the geochemical settings of the study sites, pH, O_2_, ORP and pressure in the water column collected by the RBR sensor were analyzed with non-parametric Wilcoxon tests, using R software [59]. Additionally, univariate non-parametric permutational ANOVA (PERMANOVA) analyses (PRIMER v6 and PERMANOVA+ add-on software; [60]) were performed to test for the differences in environmental variables between study sites and positions in the water column or depth in the sediment. Therefore a two-factor design was used with the fixed categorical factors ‘site’ (CO2-R, REF) and ‘position’ (80 cm, 10 cm, 0 cm, -10 cm, -20 cm asf). The effects of long-term acidified seawater conditions on *P*. *oceanica* growth characteristics (H_1_) and sessile and motile epibiont community characteristics (H_2_) were tested with PERMANOVA analyses, considering the fixed categorical factor ‘site’ (CO2-R, REF) and, in case of the motile epibionts (meiofauna), also ‘habitat’ (leaves, rhizomes). PERMANOVA analyses were based on Euclidean distance similarity measures on untransformed, and occasionally log(x+1) transformed (in case of pore water, Ωca and Ωar) univariate data, on Bray-Curtis similarity measures on standardized, square root transformed higher meiofauna taxa, nematode and copepod species, and bite mark abundances and on Jaccard similarity measures on the presence-absence data of sessile epibiont taxa. Calculation of the Pseudo-F ratio and p value (null hypothesis are rejected based on a level of significance α = 0.05) required unrestricted permutation of raw data (univariate and one-factor multivariate) or 9999 permutations of the residuals under a reduced model (multiple-factors multivariate). Posteriori pair-wise tests were conducted where significant effects were found and only when a PERMDISP test confirmed the homogeneity of multivariate dispersions. Where only a restricted number of unique permutations was possible, p-values were obtained from Monte Carlo simulations. (Dis-)similarity between and among the sessile and motile epibiont communities were visualized by non-metric multidimensional scaling (NMDS) plots (in Supporting information). The variability among and between the sessile and motile epibiont communities was quantified with SIMPER analyses on presence absence data and standardized, square root transformed abundance data, respectively (in Supporting Information). The effect of elevated *p*CO_2_ on the photosynthetic rate of *P*. *oceanica* (H_3_) was tested with a linear regression, using R software [59]. The data used for the linear regression contained no outliers and the residuals were normally distributed.

## Results

### Seawater and pore water geochemistry

**Fig 2:**
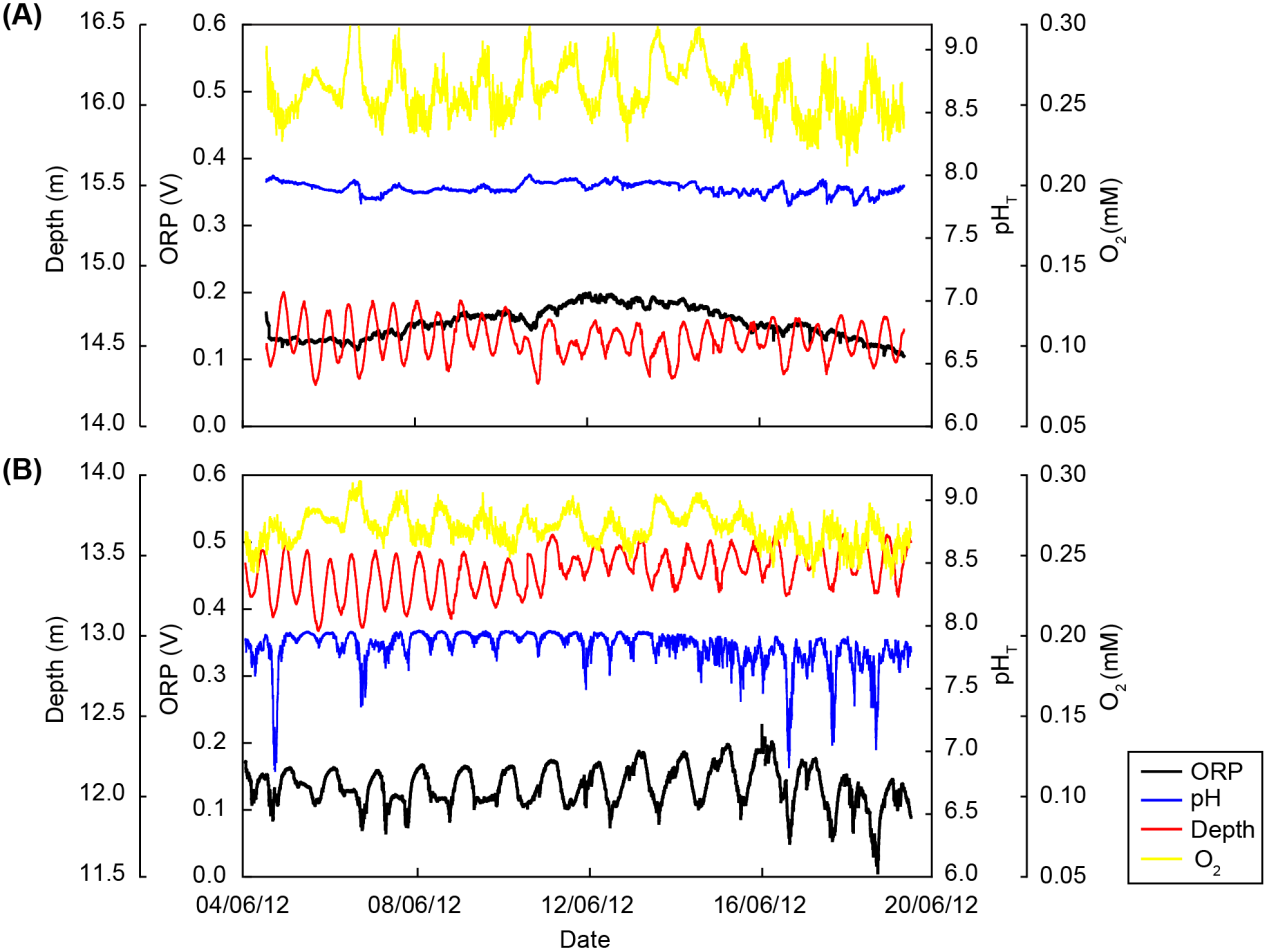
Variations in water depth, pH_T_, O_2_ and oxygen reduction potential (ORP) at both study sites. RBR sensor data were gathered over 14 to 15 days at the REF site **(A)** and the CO2-R site **(B)**. The color legend is provided in the graph.

The RBR logger data showed depth variation over time related to the tidal cycle (Fig 2). Photosynthesis can explain the observed O_2_ fluctuations at both sites. A drop in seawater pH at low tide could be observed at CO2-R, while at REF the pH co-varied with photosynthesis. Daily pH variation (ΔpH) and minima (pH_T-min_) differed between REF and CO2-R (ΔpH: 0.12 ± 0.05 and 0.49 ± 0.33, respectively; pH_T-min_: 7.75 ± 0.12 and 7.23 ± 0.97, respectively, P(perm) = 0.0001; Table 1), while daily pH maxima (pH_T-max_) did not differ (7.87 ± 0.07 and 7.86 ± 0.06, respectively). The mean seawater pH_T_ was also lower at CO2-R (7.72 ± 0.67) compared to REF (7.82 ± 0.11; Wilcoxon test, p < 2.2e-16). At CO2-R, O_2_ concentrations in the seagrass meadow reached higher values than at REF (0.268 ± 0.010 mM and 0.259 ± 0.017 mM, respectively; Wilcoxon test, p < 2.2e-16). ORP was also more variable at CO2-R (0.135 ± 0.029 mV) compared to REF (0.155 ± 0.022 mV) as a result of enhanced gas release and fluid seepage (including dissolved reductants that lowers the ORP) during low tide at CO2-R.

**Table 1:** Statistical PERMANOVA details where significant results were obtained.

	**Site**			**Position**			**Site x Position**			**Pairwise**	
	**df**	**Pseudo-F**	**%**	**df**	**Pseudo-F**	**%**	**df**	**Pseudo-F**	**%**	**Site**	**Position**
**Seawater Silicate**	1	4.997*	38.0	1	0.000106		1	0.4187		CO_2_-R > REF	
**Seawater DIC**	1	6.507*	37.3	1	0.0123		1	0.6541		CO_2_-R > REF	
**Pore water pH**	1	9.4452*	40.6	2	7.3726*	26.8	2	0.2206		REF > CO_2_-R	0 > -10 cm, -20 cm
**Pore water Ω Calcite**	1	11.384*	27.5	2	7.6073*	36.6	2	1.434		REF > CO_2_-R	0 > -20 cm
**Pore water Ω Aragonite**	1	11.3585*	27.5	2	7.6077*	36.6	2	1.434		REF > CO_2_-R	0 > -20 cm
	**Site**			**Habitat**			**Site x Habitat**			**Pairwise**	
	**df**	**Pseudo-F**	**%**	**df**	**Pseudo-F**	**%**	**df**	**Pseudo-F**	**%**	**Site**	**Habitat**
**Higher taxon composition**	1	0.692		1	16.171*	62.1	1	1.174			Leaves ≠ Rhizomes
**Nematode species composition**	1	1.8563*	13.0	1	3.4801*	24.3	1	0.9627		CO_2_-R ≠ REF	Leaves ≠ Rhizomes
**Nematode ES(51)**	1	3.368		1	22.787*	64.1	1	1.4			Rhizomes > Leaves
**Copepod species composition**	1	2.9651*	21.6	1	**1.8567**		1	0.92789		CO_2_-R ≠ REF	
**Copepod ES(51)**	1	8.270*	39.3	1	4.639		1	0.145		CO_2_-R > REF	
	**Site**									**Pairwise**	
	**df**	**Pseudo-F**	**%**							**Site**	
**pH daily variation**	1	17.071**	37.9							CO_2_-R > REF	
**pH daily minimum**	1	17.231**	38.1							REF > CO_2_-R	
**LAI**	1	16.566*	80.5							REF > CO_2_-R	
**Sessile epibiont richness**	1	4.803*	14.6							CO_2_-R > REF	
**Non-Calcifiers richness**	1	9.394*	25.1							CO_2_-R > REF	
**Sessile epibiont com. composition**	1	5.752**	17.0							CO_2_-R ≠ REF	
**Calcifiers com. composition**	1	6.310**	18.4							CO_2_-R ≠ REF	
**Non-Calcifiers com. composition**	1	5.517**	18.7							CO_2_-R ≠ REF	
**Tanaidacea density on rhizomes**	1	8.564*	68.2							CO_2_-R > REF	
**Nematoda H**_**0**_ **on rhizomes**	1	9.375*	70.1							CO_2_-R > REF	
**Copepoda H**_**0**_ **on rhizomes**	1	15.207*	79.2							CO_2_-R > REF	

The reported data include the degrees of freedom (df), Pseudo-F value, % of explained variance where significant differences were obtained and the results of the pairwise tests. (*: 0.001 ≤ p ≤ 0.05, **: p < 0.001).

Seawater characteristics based on water sampling did not differ according to the sampling position, i.e. within or above the seagrass meadows. The values were therefore averaged per site and reported in Table 2. It is important to note that seawater and pore water samples were sampled only occasionally and that therefore these snapshot results could possibly mask diurnal fluctuations. Seawater salinity was 38 at both sites and nitrite+nitrate concentrations were always below detection limit. DIC and silicate concentrations in seawater were highest at CO2-R and differed from the values measured at REF (P(perm) ≤ 0.041; Table 1). TA, pH, phosphate and ammonium, and Ωcal and Ωar in the water column were similar at both sites. Ωcal and Ωar were on average 5.5 and 3.6, respectively. Pore-water characteristics (Table 2) were generally very variable and differed with depth in the sediment between the 0 cm layer and the deepest layer (-0.2 m bsf) for pH (P(perm) ≤ 0.05), Ωcal (P(perm) = 0.0283) and for Ωar (P(perm) = 0.0281). Pore water pH, Ωar and Ωcal were lower at CO2-R compared to REF (Table 2; P(MC) ≤ 0.03; Table 1).

**Table 2:** Seawater, pore water and seagrass productivity characteristics.

		Depth bsf (m)	CO_2_-R	REF
**Seawater**	Silicate (μmol L^-1^)		1.78 ± 0.31	1.26 ± 0.37
	Phosphate (μmol L^-1^)		1.24 ± 1.56	0.46 ± 0.06
	Ammonium (μmol L^-1^)		0.27 ± 0.16	0.23 ± 0.09
	pH_T_		8.09 ± 0.69	8.11 ± 0.04
	DIC (mmol Kg^-1^)		2.23 ± 0.02	2.19 ± 0.03
	TA (mEq Kg^-1^)		2.52 ± 0.10	2.55 ± 0.02
	Ω Calcite		5.01 ± 1.62	6.02 ± 0.31
	Ω Aragonite		3.27 ± 1.06	3.92 ± 0.20
**Pore water**	Silicate (μmol L^-1^)	0	121.30 ± 196.56	2.52 ± 0.60
		0.1	37.17 ± 23.87	18.75 ± 7.06
		0.2	70.05 ± 05	21.84 ± 11.03
	Phosphate (μmol L^-1^)	0	0.71 ± 0.37	0.27 ± 0.26
		0.1	1.21 ± 0.86	1.43 ± 1.62
		0.2	1.86 ± 0.41	0.83 ± 0.36
	Ammonium (μmol L^-1^)	0	1.31 ± 2.13	1.69 ± 0.25
		0.1	2.01 ± 1.97	5.46 ± 4.01
		0.2	4.32 ±2.21	5.08 ± 5.73
	pH_T_	0	6.07 ± 1.57	8.01 ± 0.20
		0.1	5.71 ± 0.84	6.65 ±1.34
		0.2	5.57 ± 0.55	6.54 ± 1.39
	Nitrite + Nitrate (μmol L^-1^)	0	1.40 ± 0.96	1.28 ± 0.59
		0.1	1.36 ± 0.58	1.67 ± 2.04
		0.2	1.16 ± 0.72	0.44 ± 0.12
	Nitrite (μmol L^-1^)	0	0.05 ± 0.04	0.02 ± 0.02
		0.1	0.00 ± 0.00	0.06 ± 0.05
		0.2	0.00 ±.00	0.02 ± 0.02
	DIC (mmol kg^-1^)	0	3.88 ± 2.19	2.37 ± 0.17
		0.1	5.51 ± 3.23	4.19 ± 0.27
		0.2	13.52 ± 12.51	4.29 ± 0.86
	TA (mEq kg^-1^)	0	3.08 ± 0.71	2.64 ± 0.17
		0.1	3.41 ± 1.38	3.73 ± 1.01
		0.2	5.67 ± 3.76	3.62 ± 1.34
	Mn (μmol L^-1^)	0	1.91 ± 2.63	bdl
		0.1	3.54 ± 3.04	0.73 ± 0.00
		0.2	10.16 ± 12.30	0.85 ± 0.00
	Fe (μmol L^-1^)	0	32.70 ± 44.47	2.22 ± 2.32
		0.1	58.05 ± 57.86	43.00 ± 65.88
		0.2	165.51 ± 222.75	59.31 ± 92.32
	Salinity	0	38.33 ± 1.53	34.67 ± 9.24
		0.1	38.33 ± 1.53	38.67 ± 2.31
		0.2	38.00 ± 1.73	30.00 ± 8.66
	Ω Calcite	0	1.56 ± 1.83	4.76 ± 1.05
		0.1	0.46 ± 0.65	1.98 ± 1.69
		0.2	0.10 ± 0.02	1.07 ± 0.88
	Ω Aragonite	0	1.02 ± 1.20	3.10 ± 0.69
		0.1	0.30 ± 1.20	1.29 ± 1.10
		0.2	0.07 ± 0.01	0.70 ± 0.57
**Seagrass productivity**	Shoot density (N m^-2^)		674.7 ± 26.6	609.3 ± 91.8
	Leaf density (N shoot^-1^)		6.5 ± 3.2	5.5 ± 1.4
	Leaf density (N m^-2^)		4404.4 ± 930.5	3289.5 ± 196.5
	LAI (m^2^ m^-2^)		10.5 ± 0.9	18.0 ± 3.1

The seawater data were averaged over the samples collected within and above seagrass meadows, while because the values of the pore-water characteristics are highly variable with depth in the seafloor, these values are provided for the three depth positions below the seafloor surface (bsf). All data are averages ± s.d (except ± c.v. for pH_T_), bdl = below detection limit.

### *Posidonia oceanica* growth characteristics

The average shoot density (N m^-2^) and leaf density (N m^-2^ and N shoot^-1^) were highest at CO2-R (Table 2). The difference with REF was, however, not significant. Single sided surface area of leaves m^-2^ (Leaf Area Index, LAI) was higher at REF compared to CO2-R (P(MC) = 0.0161; Table 1).

### Sessile epibiont community and bite marks

The amount of CaCO_3_, a proxy for the abundance of calcifying epibionts, on the upper 5 cm of the leaves did not significantly differ between the vent site (2.54 ± 1.75 mg cm^-2^) and REF (0.74 ± 0.52 mg cm^-2^). The ratio of the total number of calcifying versus non-calcifying sessile epibiont individuals (75: 25) at REF was higher compared to the ratio at CO2-R (84: 68). This indicates an increase in non-calcifying epibiont abundance at the vent site.

The epibiont community composition was found to differ between REF and CO2-R (S1 Fig, S1 Table; P(perm) = 0.0001; Table 1). Additionally, differences occurred in both the community of calcifying and non-calcifying epibionts (P(perm) = 0.0001; Table 1). Calcifying red algae, identified to the family Corallinaceae, and *Miniacina miniacea* (a colonial sessile foraminifera) were present in a few occasions at REF and were absent from CO2-R. Most calcifying species were, however, found at both sites, and only three calcifying bryozoan species exclusively occurred at CO2-R (*Tubulipora* sp., *Cellepora* sp., *Collarina* sp.). Amongst the non-calcifiers, no species exclusively occurred at REF, while 7 species were only found at CO2-R. These shifts in community composition resulted in a higher epibiont species richness at CO2-R compared to REF (20.3 ± 7.4 and 13.0 ± 2.7, respectively, P(MC) = 0.0218; Table 1). In particular, there was a higher species richness of non-calcifying epibionts at CO2-R versus REF (CO2-R: 4.53 ± 2.83, REF: 1.67 ± 1.11, P(perm) = 0.006; Table 1), while the number of calcifying epibiont species was similar (CO2-R: 5.60 ± 2.64, REF: 5.00 ± 2.04).

Bite marks were observed on the leaves at both sites. No significant differences were found between the sites in terms of total number of bite marks (CO2-R: 0.17 ± 0.07 marks leaf^-1^, REF: 0.27 ± 0.09 marks leaf^-1^) or absolute or relative abundance of species-specific bite marks. The majority of the bite marks at the vent site were attributed to the sea urchin *Paracentrotus lividus* (80%) and the fish *Sarpa salpa* (14%), while the isopod *Idotea* sp. bite marks were only found on two occasions (6%). 96% of the bite marks at REF originated from the *P*. *lividus* and 4% from *S*. *salpa*.

### Motile epibiont (meiofauna) community

Total meiofauna density on the leaves or the rhizomes or the densities of separate meiofauna taxa did not differ between the sites (Table 3). An exception were the tanaidaceans who exclusively, though with low abundances, occurred on the rhizomes at CO2-R and not at REF (1.16 ± 0.68 ind. 10 cm^-^³, P(MC) = 0.047; Table 1). Distinct meiofaunal communities were found for the seagrass leaves and rhizomes at the higher taxon level and on nematodes at the species level (S1 Fig, S1 Table; P(perm) ≤ 0.0054; Table 1). Harpacticoid copepods were dominant in the leaf canopy, followed by nematodes, while the opposite occurred for the rhizomes (Table 3). Differences in nematode and copepod community composition were mainly attributed to shifts in the occurrences of less abundant species (i.e. < 5% relative abundance). A site effect on the community composition was only detected for nematode species (P(perm) = 0.022; Table 1). *Chromadora* sp.1 dominated at both REF and CO2-R, while a shift in relative abundances that occurred among the less dominant species changed the overall community composition. The difference found for copepod communities was attributed to non-homogeneous multivariate dispersion (Permdisp < 0.05). Meiofauna higher taxon and nematode and copepod species diversity (Hill’s indices; Table 3) on the leaves did not differ between sites. On the rhizomes, however, nematode and copepod species richness (H_0_; Table 3) was higher at CO2-R compared to REF (P(MC) ≤ 0.041; Table 1). The rarefaction index (ET(51) or ES(51)) was higher on the rhizomes compared to the leaves for nematode species, and higher at CO2-R compared to REF for the copepod species (P(perm) ≤ 0.027; Table 1). In terms of functioning, no differences in nematode trophic composition and trophic diversity were found (Table 3).

**Table 3:** Abundance and diversity of the motile epibiont (meiofauna) communities associated with *Posidonia oceanica*.

	**Meiofauna**						**Copepoda**					
	**Density**	**H**_**0**_	**H**_**1**_	**H**_**2**_	**H**_**inf**_	**ET(51)**	**Density**	**H**_**0**_	**H**_**1**_	**H**_**2**_	**H**_**inf**_	**ES(51)**
**CO2-R**	60 ± 21	10.3 ± 0.6	3.8 ± 1.1	2.5 ± 0.6	1.7 ± 0.3	7.1 ± 1.4	37 ± 14	16.0 ± 1.0	11.4 ± 0.8	8.9 ± 1.5	4.5 ± 1.3	14.9 ± 0.7
	467 ± 217	11.0 ± 2.6	3.4 ± 0.5	2.7 ± 0.2	2.0 ± 0.0	7.4 ± 1.2	43 ± 20	12.3 ± 0.6	8.3 ± 1.2	6.3 ± 1.4	3.4 ± 0.8	11.5 ± 0.5
**REF**	69 ± 33	9.7 ± 0.6	3.8 ± 1.0	2.4 ± 0.6	1.6 ± 0.2	5.2 ± 1.9	86 ± 54	10.3 ± 6.0	7.9 ± 4.3	6.5 ± 3.4	3.6 ± 1.5	10.2 ± 5.8
	169 ± 53	9.3 ± 1.5	3.6 ± 0.4	2.8 ± 0.2	2.0 ± 0.0	6.2 ± 0.5	37 ± 19	5.3 ± 3.1	4.5 ± 2.3	3.9 ± 1.7	2.5 ± 0.5	5.3 ± 3.1
	**Nematoda**											
	**Density**	**H**_**0**_	**H**_**1**_	**H**_**2**_	**H**_**inf**_	**ES(51)**	**1A**	**1B**	**2A**	**2B**	**Ɵ**^**-1**^	
**CO2-R**	6 ± 3	9.7 ± 6.7	4.7 ± 2.6	3.1 ± 1.2	1.9 ± 0.4	8.9 ± 6.0	1.7 ± 1.5	10.8 ± 11.7	76.3 ± 20.5	11.1 ± 11.7	1.7 ± 0.6	
	314 ± 174	26.0 ± 3.6	18.8 ± 3.6	13.5 ± 3.8	6.1 ± 3.1	22.8 ± 2.9	13.7 ± 6.5	7.7 ± 6.7	65.2 ± 12.5	13.4 ± 0.8	2.2 ± 0.6	
**REF**	9 ± 7	7.3 ± 0.6	5.2 ± 1.0	4.1 ± 1.4	2.5 ± 0.8	7.3 ± 0.6	0.0 ± 0.0	8.9 ± 10.7	75.0 ± 11.6	16.1 ± 12.5	1.7 ± 0.4	
	101 ± 23	16.0 ± 4.4	10.4 ± 5.5	7.6 ± 5.0	4.0 ± 2.5	15.7 ± 4.6	13.2 ± 7.6	4.8 ± 4.8	79.6 ± 11.9	2.4 ± 2.4	1.6 ± 0.4	

Density and diversity of higher taxon meiofauna, nematode and copepod species on *P*. *oceanica* leaves (grey bar) and rhizomes (white bar). Densities on the leaves and rhizomes are expressed as individuals 100 cm^-2^ and individuals 10 cm^-3^, respectively. Diversity is represented by the Hill’s indices (H_0_ –H_inf_) and the Expected number of Taxa or Species considering 51 individuals (ET(51) and ES(51), respectively). For nematode communities, also the relative abundance of feeding groups (1A, 2A, 1B, 2B) and the trophic diversity index (Ɵ^-1^) are included. The provided data are averages ± s.d.

### *Posidonia oceanica* net photosynthesis and pH profiles

**Fig 3:**
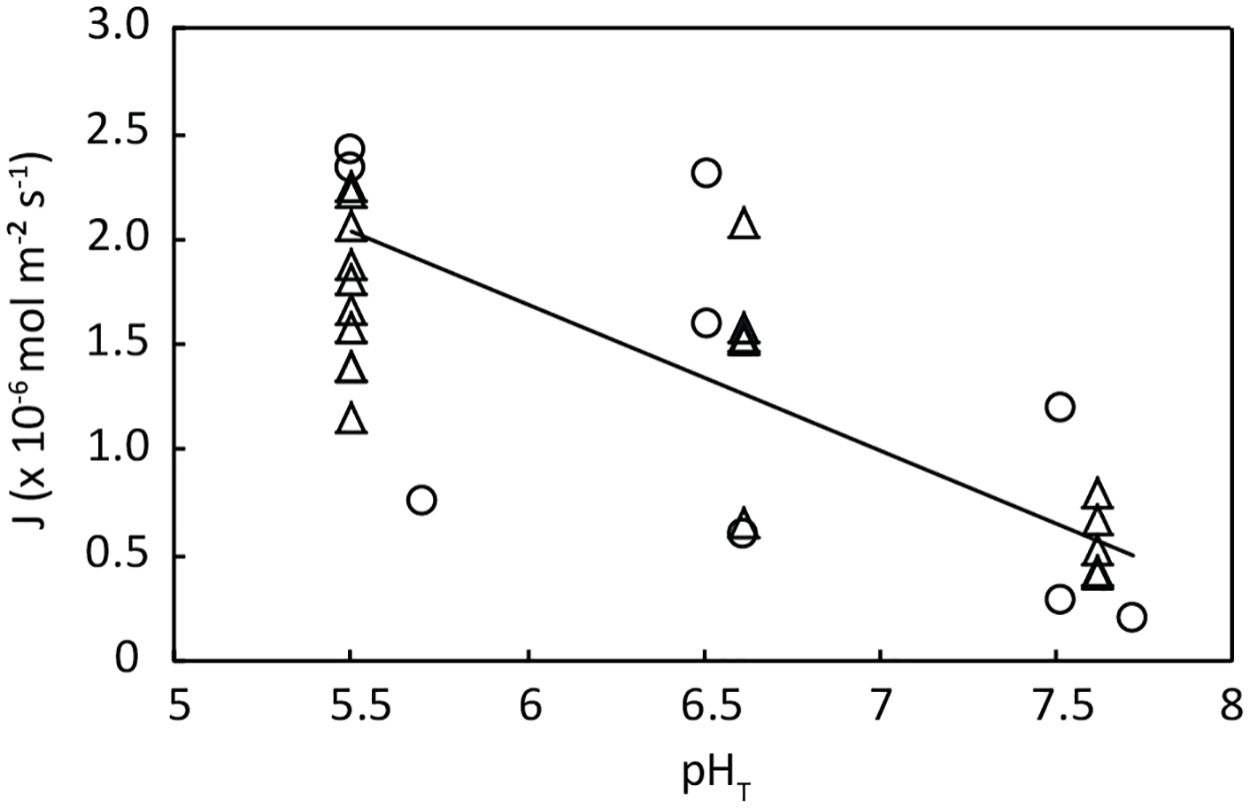
Net photosynthesis of *Posidonia oceanica* leaves subjected to seawater acidification in the laboratory experiment. Net photosynthesis (J, mol m^-2^ s^-1^) of *P*. *oceanica* was measured on leaves from REF (dots, n = 3) and CO2-R (triangles, n = 3) in relation to manipulated seawater pH_T_ (regression analysis, adjusted R^2^ = 0.50, p = 9.93e-06, no outliers, residuals normally distributed).

**Fig 4:**
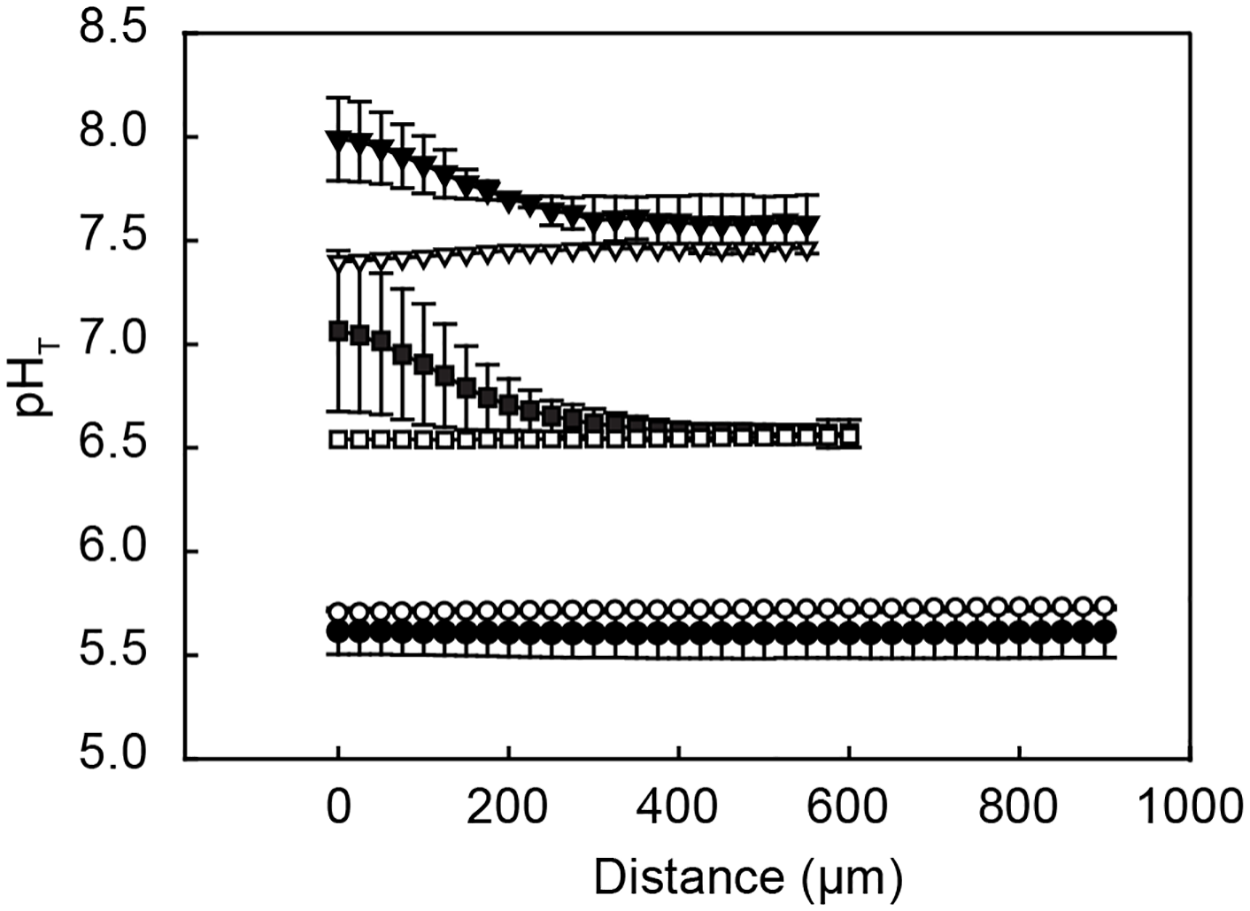
pH profiles in close vicinity of the *Posidonia oceanica* leaves (originating from REF) when subjected to seawater acidification in the laboratory experiment. pH_T_ profiles (averages ± s.d.) were measured on the convex side of *P*. *oceanica* leaves at seawater pH_T_ 7.6 (triangle), 6.6 (square) and 5.5 (circle). Measurements started from the leaf surface (distance = 0 μm), and were performed in both light (black, n = 2–3) and dark (white, n = 1) conditions.

In the laboratory experiment, we observed a trend in increasing net photosynthesis with decreasing seawater pH (adjusted R^2^ = 0.50, p = 9.93e-06; Fig 3). Moreover, under illuminated conditions, seawater pH was elevated with up to 0.4 and 0.5 units above ambient seawater values over a distance of 200μm from the leaf surface, when the seawater pH was decreased to 7.6 and 6.6, respectively (Fig 4, S2 Fig). At a seawater pH of 5.5 the pH close to the leaves was not elevated, due to strong buffering of the carbonate system when pH of the seawater approaches a pK value. Very little to no respiration was observed when leaves from REF were incubated under dark conditions as the surface pH was equal to seawater pH at all pH levels (Fig 4).

## Discussion

Typical of all highly productive ecosystems, variability in O_2_ concentrations and pH in the water centered on the *P*. *oceanica* meadows at our study site is a result of seagrass metabolic activity that follows a diurnal cycle [61]. The pH pattern at the CO_2_ vents, however, is clearly modulated by the pressured release of CO_2_ gas during low tide episodes. A similar diurnal range in pH was measured in *P*. *oceanica* meadows at CO_2_ vents off Ischia, Italy [18]. The daily pH variation at most vent sites near Ischia, however, was found to be two to three times higher when compared to pH measurements at Basiluzzo Islet vents (ΔpH: 1.0–1.6 versus 0.1–1.2, respectively). When considering the average pH values, the level of acidification in the seagrass meadows at the Basiluzzo Islet CO_2_ vents compared to the reference sites (ΔpH: 0.1) is relatively mild in comparison to the conditions found at other vent areas e.g. off Ischia (ΔpH: 0.2–0.8; [18]) and in Milne Bay, Papua New Guinea (ΔpH: 0.1–0.4 [15] or 0.4–1.5 [11]). This characteristic, together with the absence of reactive gases (H_2_S, H_2_ and CO) and organic gas compounds (CH_4_, C_2_H_6_, C_3_H_8_), the lack of temperature anomalies of vented fluids and the vicinity of a reference area with similar environmental settings (supporting data based on field studies in 2012 and 2013 available via doi:10.1594/PANGAEA.871453), makes the study area close to Basiluzzo Islet a relatively good site to study pure CO_2_ effects. The site represents a realistic, near future scenario, that conforms to ranges of predicted open ocean surface pH reductions of 0.06 to 0.32 units by 2100 [5].

Several laboratory and mesocosm experiments and in situ studies at natural CO_2_ vents have demonstrated that excessive CO_2_ increases seagrass net photosynthesis [e.g. 6–11,13]. However, particularly for *P*. *oceanica*, findings are limited and not uniform. Increasing photosynthetic rates have been measured in laboratory experiments over pH ranges from 8.2 to 6.0 (unknown pH scale; [7]) and from 9.2 to 7.9 (unknown pH scale; [28]) or at a pH_T_ of 7.3 [14]. Cox et al. [31], however, found no difference in photosynthesis during the 4 months in situ experimental eFOCE (European Free Ocean Carbon Dioxide Enrichment) incubation, where pH was lowered by 0.26 units to pH_T_ of 7.7. Also when considering photosynthetic efficiency and electron transport rates at the CO_2_ vents off Ischia (mean pH_T_: 8.2 versus 7.6; [18]) or in a laboratory experiment that simulated different levels of OA (380, 750 and 1000 ppm pCO_2_) over 1 to 3 weeks [23], no effects were found. Our laboratory experiment revealed an increasing trend in *P*. *oceanica* net photosynthesis with *p*CO_2_ increasing and pH decreasing over a pH_T_ range of 7.6 to 5.5. Taking into account the reduced LAI at the vent site, it is unlikely that the higher O_2_ concentration measured in the meadows at CO2-R compared to REF are solely attributed to seagrass photosynthesis. Johnson et al. [62] observed an increase in epiphytic diatom abundance on artificial substrata placed along a CO_2_ gradient at the vent area off Vulcano Island (median pH_NBS_: 8.2–7.7). Diatom abundance at the water-sediment interface in a shallow subtidal area in the Adriatic Sea was also found to be linked to the availability of silicate [63]. Likewise, diatom abundance on the seagrass leaves at the vents off Basiluzzo may have been stimulated by increased CO_2_ and silicate concentrations, the latter being released by the weathering of sediments. Their potential contribution to higher O_2_ concentrations at the vent site is, however, purely speculative as we didn’t quantify the epiphytic diatom abundance.

The overgrowth of seagrass leaves with algal epiphytes can initiate a negative growth response of seagrasses [25,64] by reducing the quantity and quality of light reaching the leaves. The overall plant performance during day-time is thus impeded, while an enhanced diffusive boundary layer reduces the exchange of gasses and nutrients with the ambient water column [64]. This combined with the stimulation of seagrass growth, may explain the elevated *P*. *oceanica* shoot density at the CO_2_ vent site off Ischia when epiphytic coverage was severely reduced (pH_T-mean_: 7.60, pH_T-min_: 6.98; [18]). Under less severely acidified conditions at Ischia (pH_T-min_: 8.15–7.67), seagrass shoot density did not increase, though epiphyte coverage gradually decreased [18]. The latter is partly in agreement with the findings near Basiluzzo Islet, where at average pH_T-min_ of 7.23, shoot and leaf density also remained unaffected, despite an increase in non-calcareous epibiont abundance. It must be said that shoot density at REF had a relatively high variability compared to shoot densities of *P*. *oceanica* reported by Hall-Spencer et al. [18] and Balestri et al. [65] for the NW Mediterranean Sea. Therefore, we cannot exclude an effect of low replication in our study.

The significantly higher LAI, which indicates longer leaves, at REF compared to CO2-R corresponds to previous findings in the *P*. *oceanica* meadows at the CO_2_ vents off Ischia [34]. The increased leaf erosion at Ischia was attributed to various grazers. Likewise, *Cymodocea nodosa*, also known as little Neptune seagrass, exposed to higher *p*CO_2_ at the Vulcano Island vent area were found to be more prone to grazing due to a reduction of phenolic substances in the seagrass leaves [24]. We found no evidence of increased grazing based on the number of bite marks at Basiluzzo Islet. The dominant grazer, the sea urchin *Paracentrotus lividus*, prefers leaves covered with epibiota [66] and adult, thicker leaves [67], and Cox et al. [31] did measure an increase in *P*. *oceanica* leaf thickness after 4 months in-situ exposure to acidified conditions (pH lowered with 0.26 units to 7.7). Therefore, it could be that grazing at CO2-R was stimulated by the higher abundance of sessile non-calcifying epibionts we observed and perhaps an increased thickness of the leaves at the vent site, and that leaf tips eroded after fragmentation was triggered by consumption. Specific statements about the susceptibility of seagrasses to grazing are clearly not possible based on the number of bite marks only and require controlled cage experiments.

The CO_2_ vent conditions at Basilluzo Islet induced a shift in the sessile epibiont community composition, for both the calcifying and the non-calcifying group of organisms. Among the calcifiers, only the coralline red algae were obviously absent at the vent site. Alternatively, some calcifying bryozoan species (*Tubulipora* sp., *Cellepora* sp., *Collarina* sp.) appeared to benefit from the more acidic conditions and consequently the release of competitive pressure from formerly occurring or dominant species, as they exclusively occurred at the vent site. A similar community shift was also observed on *P*. *oceanica* leaves at the CO_2_ vents off Ischia (pH_T-mean_: 7.7 [4]) and following a 2- and 4-week incubation of *P*. *oceanica* leaves in the laboratory at pH_T_ 7.0 [4] and pH_T_ 7.7 and 7.3 [14], respectively. Likewise, the diversity of crustose coralline algae that live on *Enhalus acoroides*, aslo known as tape seagrass, was reduced under the influence of the acidified conditions at vent sites in Papua New Guinea (pH_T-median_: 7.8 [36]). The high vulnerability of coralline algae versus the relatively high tolerance of at least some bryozoan species is related to the difference in their skeleton mineralogy. The relatively lower Mg content in the calcium carbonate skeleton of bryozoans makes them more resistant to chemical dissolution at low pH [68]. At Basiluzzo Islet, the shift in the sessile epibiont community composition did, however, not affect the total abundance of calcifying epibionts; evidenced by the lack of variation in CaCO_3_ weight. Likewise, no change in CaCO_3_ mass and in percent coverage of invertebrate calcifiers and crustose coralline algae on *P*. *oceanica* occurred after 4 months of exposure to reduced pH conditions (pH_T-mean_: 7.75) in the FOCE system of Cox et al. [33] (NW Mediterranean Sea). Moreover, the exclusive occurrence of a number of sessile epibiont taxa at the CO_2_ vents near Basiluzzo Islet even resulted in an overall higher species richness under acidified conditions. Both these results contrast with findings from earlier studies on epibionts associated with natural and mimic *P*. *oceanica* seagrasses and artificial collector units, and studies on other benthic plant components and animals on hard substrata at the CO_2_ vent area off Ischia [18,34,69–71]. At Ischia, a general reduction in the abundance or a total disappearance of calcifying forms resulted in a drop in species number at the low pH stations (mean pH_T_ varied from 6.6 to 7.4). Hall-Spencer et al. [18] indicated that the organisms with aragonite skeletons were absent at mean Ωar ≤ 2.5, which is a value lower than measured at our study sites (mean Ωar = 3.9 ± 0.7). Similarly, the percent cover of epiphytic calcareous algae decreased while fleshy macroalgal cover increased, though less substantial, from control towards vent sites in Milne Bay, Papua New Guinea (median Ωar = 3.5 versus 2.9 [15]). Similarly, in a laboratory experiment Cox et al. [14] observed a decrease of the carbonate content in epiphytes on *P*. *oceanica* under corrosive conditions (Ωar = 0.5–0.8) at pH_T_ 7.32. Donnarumma et al. [34] reported that the implanted *P*. *oceanica* mimics at the CO_2_ vent sites off Ischia were dominated by filamentous algae and non-calcareous taxa such as hydroids and tunicates, which resulted in a poor and simplified assemblage. The relatively mild acidification effects observed at our study site, where the average pH_T_ at the CO_2_ vent site is 7.72, which is 0.1 units lower compared to the reference site, suggests that the sessile epibiont communities can cope with a certain degree of OA. However, these results should be interpreted with caution because of the inherent natural variability in *p*CO_2_, pH and Ωar at shallow natural vents, the influences from non-acidified surrounding areas (e.g. recruitment) and incomplete understanding of historical biochemistry [72]. The study of Cox et al. [33], however, where a portion of *P*. *oceanica* meadows was enclosed and exposed to control and acidified conditions (pH_T-mean_: 8.01 vs. 7.75) for 4 months, also concludes that negative impacts from OA on epiphytic communities were smaller than expected.

Under natural conditions, Mediterranean *P*. *oceanica* meadows are able to modify pH in the water column up to 0.5 pH units diurnally through photosynthetic activity and community metabolism [28,30]. Calcifying organisms may benefit from the modification of the carbonate system by these meadows as they provide a daily window of maximum Ωar where calcification is more cost efficient [30]. This protective or buffering capacity might, however, be at risk when the health of seagrass meadows is altered by pressing environmental stressors [30]. Apart from the unaltered leaf and shoot density and the increase in net photosynthesis at the CO_2_ vents off Basiluzzo Islet, the results from this study temper this concern, at least for the effect related to ocean acidification. The pH profiles measured on *P*. *oceanica* leaves in our laboratory experiment revealed that the exposure of epibionts to ambient acidified seawater conditions is restricted to the hours when no photosynthesis occurs. Under dark conditions the pH at the leaf surface equaled ambient seawater, as seagrass leaf respiration was very low. Seagrass photosynthesis buffered the leaf surface under illuminated conditions in the laboratory experiment. This in turn created a microenvironment where pH is elevated 0.2–0.4 to 0.3–0.5 units where ambient seawater pH_T_ was lowered to 7.6 and 6.6. This period may be long enough and occurring often enough so that certain sessile or motile species can maintain calcification and/or normal metabolism. The diffusive boundary layer (DBL) in our experiments was 200 μm thick. The DBL may vary in nature depending on the leaf epiphyte cover or other factors that affect hydrodynamics. Based on our findings, we hypothesize that the thickness of the DBL and the periodical exposure to pH minima and *p*CO_2_ maxima, primarily determine the observed biotic responses at CO_2_ vents.

Several studies have demonstrated that the abundance and diversity of dominant motile invertebrates or meiofauna on marine vegetation are positively correlated with habitat complexity. This habitat complexity is measured by e.g. the biomass of epiphytic algae, percentage of leaf coverage and epiphyte structure, or seagrass density [37,73]. At sites where moderate CO_2_ seepage occurs at Basiluzzo Islet, more non-calcifying sessile epibionts and a higher overall sessile epibiotic species richness were detected compared to the reference site. This suggests a potentially more complex seagrass ecosystem when the surrounding seawater pH is decreased. This could explain the higher copepod diversity at CO2-R compared to REF. Conversely, no difference in the density and diversity of total meiofauna, nematodes in particular, was found between the sites. Also in terms of community composition, no major changes were detected on higher taxon level of meiofauna or on nematodes and copepods at the species level. These results suggest that meiofauna have little physiological intolerance to the low pH they are exposed to for at least part of the day. Garrard et al. [35] suggests the same for macro-invertebrates living in *P*. *oceanica* seagrass meadows at a CO_2_ vent off Ischia (pH_T_ range: 8.2–7.1) after detecting increased abundances and unchanged diversity at the vent site. At Basiluzzo Islet, distinct differences in meiofauna community composition occurred between the leaves and rhizomes, which agrees with earlier findings on *P*. *oceanica* in non-acidified conditions near Ischia [37] and Corsica [74]. Similarly, the highest total meiofauna density was found on the rhizomes and harpacticoid copepods dominated the meiofauna community in the leaf region [37,74]. Additionally, on the functional level of nematode communities, no site effect was detected at Basiluzzo Islet. Epistratum feeders, i.e. the group of nematodes that scrape the biofilm from surfaces or puncture and empty diatoms or microalgae [56], dominated at both sites, and on both seagrass parts with ≥ 65% relative abundance. This finding supports earlier suggestions of a close link with microbe and diatom based food chains which were based on the relationship between the abundance or biomass of nematodes and bacteria or diatoms on *P*. *oceancia* [37,75]. Similarly, based on stable isotope signatures, epiphytic biofilms have also been shown to contribute 70% of the diet of copepods living in close association with *P*. *oceanica* leaves [74]. The success of invertebrates in tolerating a decrease in pH in seagrass meadows is suggested to be partly related to the high abundance of food. This may compensate the metabolic cost of OA tolerance [35].

## Conclusions

The CO_2_ vent area at Basiluzzo Islet has allowed us to study the response of *P*. *oceanica* seagrass ecosystems to a level of OA that falls in the range of seawater pH reductions predicted to generally prevail by the year 2100. Despite the inherent short-comings of natural CO_2_ vents as analogues for OA and the limited replication in our study we hypothesize that the primary determinants of the observed biotic responses at CO_2_ vents may be the buffering capacity by the photosynthesis of *P*. *oceanica*, the thickness of the DBL, and the periodical exposure to pH minima and *p*CO_2_ maxima. Our findings suggest that with an average pH decrease of 0.1 units in seawater, changes in *P*. *oceanica* meadows will mainly occur on the level of the seagrass sessile epibiont community composition. However, *P*. *oceanica* seagrass productivity above the seafloor surface, net epibiont calcification, meiofauna community composition, abundance and function, and overall biodiversity will remain stable. The shorter leaves found at the vent sites, nevertheless, do seem to indicate an increased vulnerability to erosion, potentially by herbivores.

Although our study illustrated the relevance of an integrative ecosystem-based approach in studying long-term effect of OA at a natural analogue, it is likely that OA is not occurring in isolation and that biological responses may differ with the occurrence of additional stressors. Studying the effects of OA and potential interacting global stressors, such as temperature and oxygen, are restricted to short to medium-term laboratory experiments, but should ideally be supported by mesocosm (e.g. FOCE) and field studies at appropriate natural laboratories. Moreover, studies at natural laboratories should incorporate sufficient replication and monitoring of physico-chemical parameters on an annual scale to ensure that the biological responses observed during a short period reflect long-term dynamics of these parameters.

## Supporting information

S1 Fig Community structures of *P*. *oceanica* epibionts. Non-metric multidimensional scaling (NMDS) ordination plots representing the community compositions of sessile epibiont taxa (A, 2D stress: 0.21), meiofauna at higer taxon level (B, 2D stress: 0.06), copepod species (C, 2D stress: 0.09) and nematode species (D, 2D stress: 0.12). The numbers provided to epibiont subsamples refer to the respective replicates (1 to 3). The percentage of similarity (SIMPER) is provided for each subset of samples.
(TIF)

S2 Fig pH profiles in close vicinity of the *Posidonia oceanica* leaves (originating from CO2-R) when subjected to seawater acidification. pH_T_ profiles (averages ± s.d.) were measured on the convex side of P. oceanica leaves at seawater pH 7.6 (triangle), 6.6 (square) and 5.5 (circle). Measurements started from the leaf surface (distance = 0 μm), and were performed in light (black, n = 3) conditions.
(TIF)

S1 Table Relative abundance of meiofauna, copepod, nematode and sessile epibiont taxa on the *Posidonia oceanica* rhizomes and leaves and in the vent site and reference site. Meiofauna, copepod and nematode data are based on abundances, sessile epibiont data are based on presence/absence data.
(DOCX)
