# Supplementary figures and images for: Response of Posidonia oceanica seagrass and its epibiont communities to ocean acidification
Source: PLoS One. 2017 Aug 9;12(8):e0181531. doi: 10.1371/journal.pone.0181531 (PMC5549886; doi:10.1371/journal.pone.0181531)

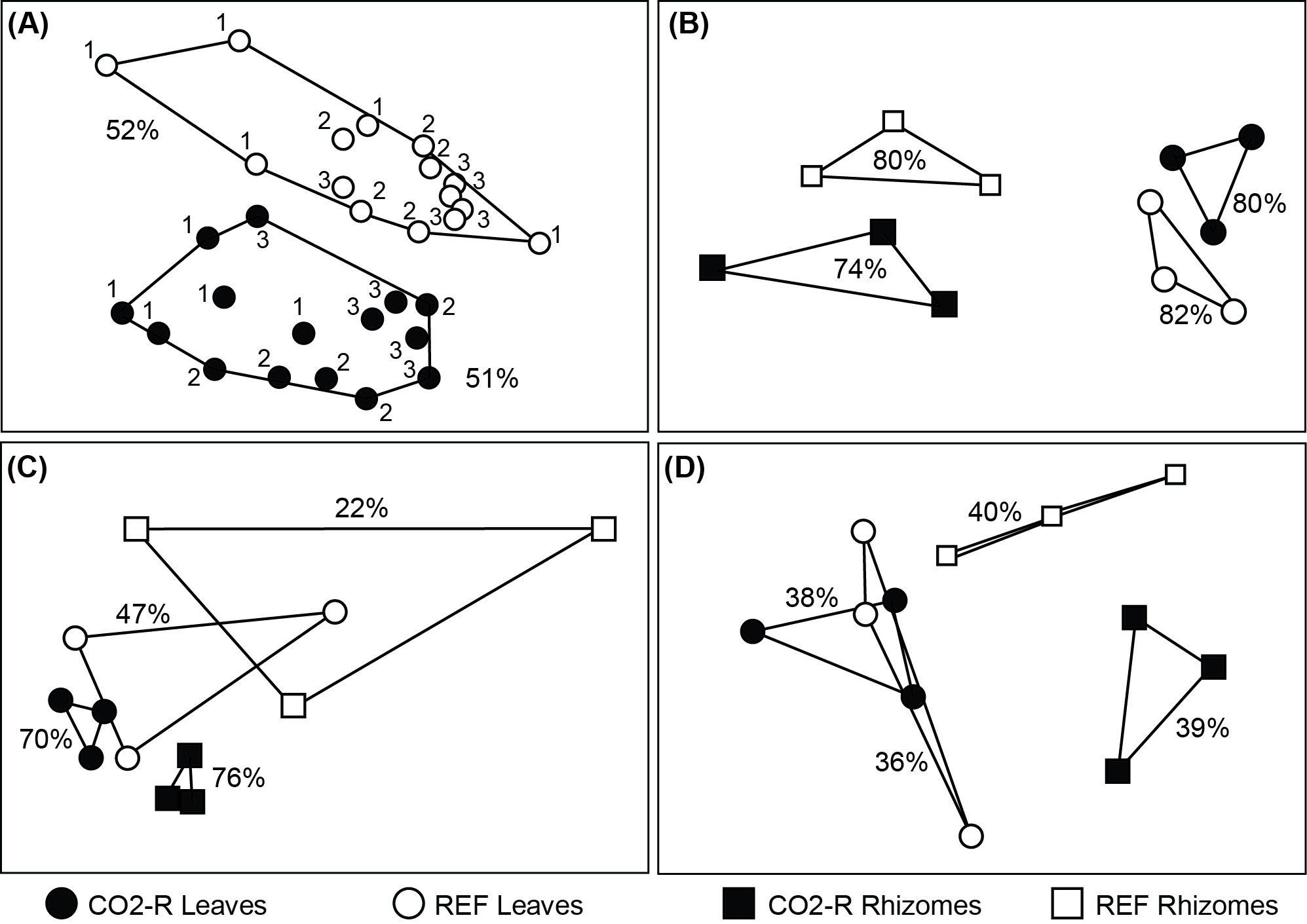

Supplement: S1 Fig — Non-metric multidimensional scaling (NMDS) ordination plots representing the community compositions of sessile epibiont taxa (A, 2D stress: 0.21), meiofauna at higer taxon level (B, 2D stress: 0.06), copepod species (C, 2D stress: 0.09) and nematode species (D, 2D stress: 0.12). The numbers provided to epibiont subsamples refer to the respective replicates (1 to 3). The percentage of similarity (SIMPER) is provided for each subset of samples. (TIF) [file pone.0181531.s001.tif]

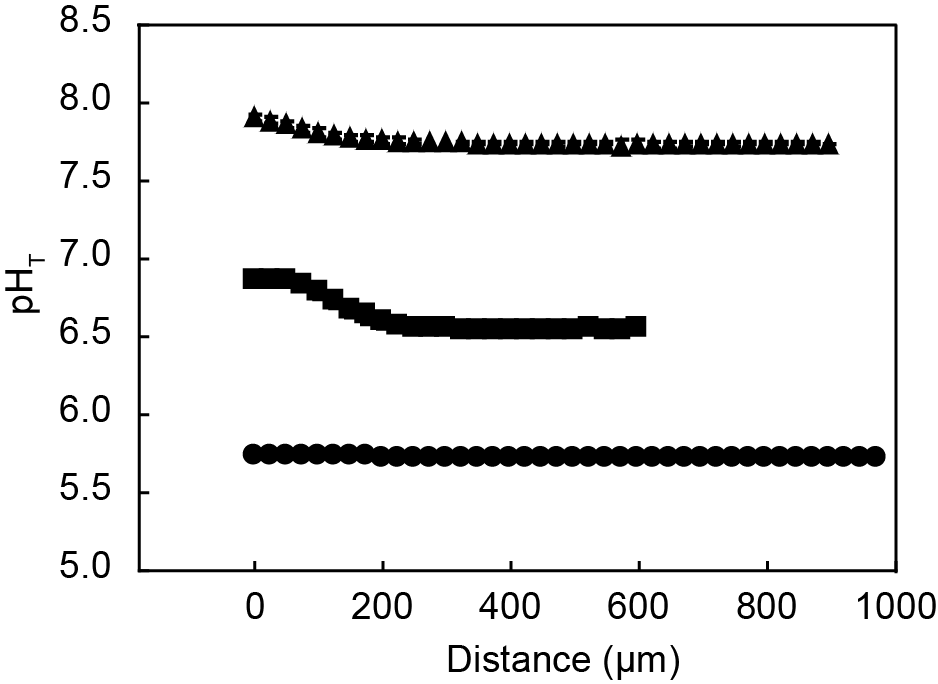

Supplement: S2 Fig — pHT profiles (averages ± s.d.) were measured on the convex side of P. oceanica leaves at seawater pH 7.6 (triangle), 6.6 (square) and 5.5 (circle). Measurements started from the leaf surface (distance = 0 μm), and were performed in light (black, n = 3) conditions. (TIF) [file pone.0181531.s002.tif]
