# Supplementary material for: Response of Posidonia oceanica seagrass and its epibiont communities to ocean acidification
Source: PLoS One. 2017 Aug 9;12(8):e0181531. doi: 10.1371/journal.pone.0181531 (PMC5549886; doi:10.1371/journal.pone.0181531)
Supplement: S1 Table — Meiofauna, copepod and nematode data are based on abundances, sessile epibiont data are based on presence/absence data. (DOCX) [file pone.0181531.s003.docx]

|  | **Meiofauna** |  |  | **Copepoda** |  |  | **Nematoda** |  |  | **Sessile epibionts** |  |
| --- | --- | --- | --- | --- | --- | --- | --- | --- | --- | --- | --- |
|  |  | **%** |  |  | **%** |  |  | **%** |  |  | **%** |
| **Leaves** | Copepoda | 62.4 ± 8.3 |  | *Ectinosoma dentatum* | 17.2 ± 8.7 |  | *Chromadora* sp. 1 | 45.2 ± 19.6 |  |  |  |
|  | Nematoda | 11.6 ± 4.2 |  | *Tisbe ensifer* | 14.6 ± 11.1 |  | *Loveninema unicornis* | 7.0 ± 8.5 |  |  |  |
|  | Nauplii | 9.3 ± 4.7 |  | *Ameira minuta* | 13.5 ± 13.6 |  | *Desmodora* sp. 1 | 5.6 ± 13.6 |  |  |  |
|  | Polychaeta | 5.0 ± 2.2 |  | *Ameira longipes* | 9.5 ± 5.3 |  | *Prochromadorella* sp. 1 | 5.5 ± 7.0 |  |  |  |
|  | Gastropoda | 4.0 ± 1.8 |  | *Amphiascus* sp. 4 | 7.2 ± 6.0 |  | *Syringolaimus* sp. 1 | 5.3 ± 4.5 |  |  |  |
|  | Ostracoda | 2.3 ± 2.8 |  | *Amphiascus* sp. 2 | 6.0 ± 6.7 |  | *Camacolaimus* sp. 1 | 5.0 ± 12.3 |  |  |  |
|  | Amphipoda | 1.7 ± 1.3 |  | *Porcellidium* sp. 1 | 4.8 ± 4.4 |  | *Symplocostoma* sp. 1 | 4.2 ± 5.3 |  |  |  |
|  | Halacaroidea | 1.4 ± 1.4 |  | *Porcellidium ovatum* | 3.6 ± 2.7 |  | *Phanoderma* sp. 1 | 3.7 ± 8.0 |  |  |  |
|  | Isopoda | 0.8 ± 0.3 |  | *Microsetella* sp. 1 | 3.6 ± 8.1 |  | *Chromadorina* sp. 1 | 2.9 ± 7.2 |  |  |  |
|  | Turbellaria | 0.5 ± 0.9 |  | *Tisbe furcata* | 3.2 ± 2.7 |  | *Euchromadora* sp. 1 | 2.9 ± 3.3 |  |  |  |
|  |  |  |  | *Porcellidium viride* | 2.7 ± 3.7 |  | *Pontonema* sp. 1 | 2.1 ± 4.0 |  |  |  |
|  |  |  |  | *Amphiascus* sp. 3 | 2.5 ± 2.5 |  |  |  |  |  |  |
|  |  |  |  | *Amphiascus* sp. 1 | 2.3 ± 2.6 |  |  |  |  |  |  |
|  |  |  |  |  |  |  |  |  |  |  |  |
| **Rhizomes** | Nematoda | 62.8 ± 8.9 |  | *Ameira longipes* | 27.5 ± 14.8 |  | *Chromadora* sp. 1 | 16.6 ± 18.3 |  |  |  |
|  | Copepoda | 20.7 ± 6.9 |  | *Ameira minuta* | 14.3 ± 18.8 |  | *Prochromadorella* sp. 1 | 10.2 ± 7.5 |  |  |  |
|  | Polychaeta | 7.4 ± 5.3 |  | *Ectinosoma dentatum* | 8.4 ± 6.0 |  | *Desmodora* sp. 1 | 5.7 ± 9.7 |  |  |  |
|  | Nauplii | 3.2 ± 2.6 |  | *Porcellidium tenuicauda* | 8.3 ± 20.4 |  | *Paracanthonchus* sp. 1 | 5.1 ± 3.8 |  |  |  |
|  | Ostracoda | 1.5 ± 1.4 |  | *Amphiascus* sp. 4 | 7.3 ± 6.2 |  | *Microlaimus* sp. 1 | 4.7 ± 4.6 |  |  |  |
|  | Halacaroidea | 1.2 ± 0.8 |  | *Amphiascus* sp. 2 | 6.8 ± 7.8 |  | *Acantholaimus* sp. 1 | 4.2 ± 8.9 |  |  |  |
|  | Amphipoda | 1.1 ± 1.3 |  | *Laophonte cornuta* | 4.8 ± 5.8 |  | *Desmodora* sp. 2 | 3.0 ± 2.6 |  |  |  |
|  | Gastropoda | 0.8 ± 1.7 |  | *Amphiascus* sp. 3 | 4.3 ± 6.0 |  | *Endeolophos* sp. 1 | 2.8 ± 3.8 |  |  |  |
|  | Oligochaeta | 0.5 ± 0.7 |  | *Tisbe ensifer* | 3.6 ± 6.1 |  | *Xenella* sp. 1 | 2.5 ± 4.8 |  |  |  |
|  |  |  |  | *Amphiascus* sp. 1 | 3.4 ± 3.9 |  | *Chromadorina* sp. 1 | 2.4 ± 6.0 |  |  |  |
|  |  |  |  | *Tegastes satyrus* | 2.6 ± 2.6 |  | *Spilophorella* sp. 1 | 2.4 ± 2.3 |  |  |  |
|  |  |  |  |  |  |  | *Camacolaimus* sp. 1 | 2.1 ± 4.1 |  |  |  |
|  |  |  |  |  |  |  |  |  |  |  |  |
| **REF** |  |  |  | *Ameira minuta* | 21.7 ± 19.9 |  | *Chromadora* sp. 1 | 32.0 ± 21.4 |  | *Hydrolithon farinosu* (red alga) | 26.8 |
|  |  |  |  | *Ameira longipes* | 18.0 ± 15.4 |  | *Prochromadorella* sp. 1 | 12.9 ± 6.9 |  | *Aetea* sp. (bryozoan) | 16.3 |
|  |  |  |  | *Ectinosoma dentatum* | 15.6 ± 11.3 |  | *Camacolaimus* sp. 1 | 5.4 ± 12.1 |  | Algae indet. (filamenteous algae) | 14.1 |
|  |  |  |  | *Porcellidium tenuicauda* | 8.3 ± 20.4 |  | *Chromadorina* sp. 1 | 5.4 ± 8.4 |  | *Chorizopora* sp. (bryozoan) | 13.6 |
|  |  |  |  | *Tisbe ensifer* | 7.1 ± 8.8 |  | *Phanoderma* sp. 1 | 3.7 ± 8.0 |  | *Cellepora* sp. (bryozoan) | 11.0 |
|  |  |  |  | *Microsetella* sp. 1 | 4.4 ± 8.1 |  | *Paracanthonchus* sp. 1 | 3.1 ± 4.5 |  | *Electra* sp. (bryozoan) | 6.4 |
|  |  |  |  | *Amphiascus* sp. 4 | 4.0 ± 6.2 |  | *Syringolaimus* sp. 1 | 3.1 ± 4.8 |  | cf. Cheilostomatida indet. (bryozoan) | 4.8 |
|  |  |  |  | *Laophonte cornuta* | 3.7 ± 6.3 |  | *Thalassomonhystera* sp. 1 | 2.9 ± 3.3 |  | *Mimosella* sp. (bryozoan) | 3.9 |
|  |  |  |  | *Tisbe furcata* | 2.7 ± 3.2 |  | *Endeolophos* sp. 1 | 2.6 ± 4.0 |  |  |  |
|  |  |  |  | *Amphiascus* sp. 2 | 2.4 ± 4.8 |  | *Loveninema unicornis* | 2.5 ± 5.0 |  |  |  |
|  |  |  |  | *Sacodiscus littoralis* | 2.1 ± 2.7 |  | *Xenella* sp. 1 | 2.0 ± 5.0 |  |  |  |
|  |  |  |  |  |  |  |  |  |  |  |  |
| **CO2-R** |  |  |  | *Ameira longipes* | 18.9 ± 14.4 |  | *Chromadora* sp. 1 | 29.8 ± 27.4 |  | *Hydrolithon farinosum* (Red alga) | 21.9 |
|  |  |  |  | *Tisbe ensifer* | 11.2 ± 12.1 |  | *Desmodora* sp. 1 | 11.2 ± 14.3 |  | *Lichenopora* sp. (bryozoan) | 21.9 |
|  |  |  |  | *Amphiascus* sp. 4 | 10.6 ± 3.2 |  | *Loveninema unicornis* | 4.9 ± 8.4 |  | *Botryllus* sp. (tunicate) | 12.5 |
|  |  |  |  | *Amphiascus* sp. 2 | 10.3 ± 6.8 |  | *Acantholaimus* sp. 1 | 4.6 ± 8.7 |  | *Aetea* sp. (bryozoan) | 9.1 |
|  |  |  |  | *Ectinosoma dentatum* | 10.0 ± 3.4 |  | *Syringolaimus* sp. 1 | 3.8 ± 3.4 |  | *Mimosella* sp. (bryozoan) | 5.5 |
|  |  |  |  | *Amphiascus* sp. 3 | 6.2 ± 4.8 |  | *Microlaimus* sp. 1 | 3.3 ± 4.0 |  | Algae indet. (filamenteous algae) | 5.2 |
|  |  |  |  | *Ameira minuta* | 6.1 ± 4.2 |  | *Symplocostoma* sp. 1 | 2.8 ± 5.5 |  | *Microporella* sp. (bryozoan) | 4.8 |
|  |  |  |  | *Amphiascus* sp. 1 | 4.2 ± 3.3 |  | *Prochromadorella* sp. 1 | 2.8 ± 3.0 |  | Aglaopheniidae indet. (hydrozoan) | 3.5 |
|  |  |  |  | *Porcellidium* sp. 1 | 4.1 ± 4.2 |  | *Spilophorella* sp. 1 | 2.5 ± 2.7 |  | *Chorizopora* sp. (bryozoan) | 3.3 |
|  |  |  |  | *Porcellidium viride* | 2.7 ± 3.7 |  | *Paracanthonchus* sp. 1 | 2.3 ± 3.0 |  | cf. Cheilostomatida indet. (bryozoan) | 2.4 |
|  |  |  |  | *Lourinia armata* | 2.7 ± 2.2 |  | *Euchromadora* sp. 1 | 2.1 ± 2.9 |  |  |  |
|  |  |  |  | *Tegastes satyrus* | 2.2 ± 2.0 |  | *Acanthopharynx* sp. 1 | 2.1 ± 1.8 |  |  |  |
|  |  |  |  | *Porcellidium ovatum* | 2.1 ± 2.5 |  |  |  |  |  |  |
